# Efficacy and safety of iguratimod combined with methotrexate vs. methotrexate alone in rheumatoid arthritis: A systematic review and meta-analysis of randomized controlled trials

**DOI:** 10.1007/s00393-020-00944-7

**Published:** 2020-12-21

**Authors:** L.-J. Chen, Y.-J. Zhou, Z.-H. Wen, F. Tian, J.-Y. Li

**Affiliations:** 1 Department of Rheumatology and Immunology, The Affiliated ZhuZhou Hospital of XiangYa Medical College, Central South University, 116 South Changjiang Road, 412007 ZhuZhou, Hunan Province China; 2 Department of Neurosurgery, Union Hospital, Tongji Medical College, Huazhong University of Science and Technology, 430022 Wuhan, China

**Keywords:** Iguratimod, Methotrexate, Rheumatoid arthritis, Meta-analysis, Randomized controlled trials, Iguratimod, Methotrexat, Rheumatoide Arthritis, Metaanalyse, Randomisierte kontrollierte Studie

## Abstract

The current systematic review and meta-analysis aims to evaluate the efficacy and safety of iguratimod (IGU) combined with methotrexate (MTX) versus MTX alone in rheumatoid arthritis (RA). Two independent investigators searched for original randomized controlled trials (RCTs) related to the combination of IGU and MTX in RA published before November 1, 2019, in PubMed, Cochrane Library, Embase, the China National Knowledge Infrastructure (CNKI), the Chinese Biomedical Literature Database (CBM), and WanFang Data. Additionally, we searched clinical trial registry websites. We assessed the methodological quality of the included trials using the Cochrane Collaboration tool and the seven-point Jadad scale. Statistical analyses were performed using Review Manager (RevMan) 5.3 (Copenhagen: The Nordic Cochrane Centre, The Cochrane Collaboration, 2014). Meta-regression and publication bias analyses were performed using Stata version 14 software (StataCorp., College Station, TX, USA). A total of 7 RCTs consisting of 665 participants, with 368 participants in the active arm and 297 in the placebo arm, were included in the meta-analysis. The American College of Rheumatology (ACR) value was better in the IGU + MTX group than in the MTX alone group, with a pooled relative risk (RR) for ACR20 (American College of Rheumatology 20% improvement criteria), ACR50, and ACR70 of 1.40 (95% CI, 1.13–1.74), 2.09 (95% CI, 1.67–2.61), and 2.24 (95% CI, 1.53–3.28), respectively. The results of the meta-analysis demonstrated that there was no statistical significance in adverse events (1.06 (95% CI, 0.92–1.23)). The combined treatment is an effective, safe, and economical treatment option for patients who do not respond well to methotrexate alone or for patients who cannot afford expensive biologics that have no confirmed efficacy.

## Abstract (de)

Ziel der vorliegenden systematischen Übersichtsarbeit und Metaanalyse war es, die Wirksamkeit und Sicherheit von Iguratimod (IGU) in Kombination mit Methotrexat (MTX) versus MTX allein bei rheumatoider Arthritis (RA) zu untersuchen. Dazu suchten 2 unabhängige Untersucher in den Datenbanken PubMed, Cochrane Library, Embase, China National Knowledge Infrastructure (CNKI), Chinese Biomedical Literature Database (CBM) und WanFang Data nach Originalpublikationen randomisierter kontrollierter Studien (RCT) hinsichtlich der Kombination von IGU und MTX bei RA, die vor dem 1. November 2019 publiziert worden waren. Zusätzlich wurden Websites zu klinischen Studienregistern durchsucht. Die methodische Qualität der einbezogenen Studien wurde anhand des Bewertungsschemas der Cochrane Collaboration und der 7 Punkte umfassenden Jadad-Skala beurteilt. Statistische Analysen wurden unter Einsatz des Programms Review Manager (RevMan) 5.3 (The Nordic Cochrane Centre, Kopenhagen, The Cochrane Collaboration, 2014) durchgeführt. Metaregressions- und Publikationsbiasanalysen wurden unter Verwendung der Software Stata, Version 14 (Fa. StataCorp., College Station, TX, USA) durchgeführt. In die Metaanalyse einbezogen wurden 7 RCT mit 665 Teilnehmern, davon 368 im aktiven Studienarm und 297 im Placeboarm. Der Score gemäß American College of Rheumatology (ACR) war in der Gruppe IGU + MTX besser als in der Gruppe mit MTX allein, das gepoolte relative Risiko (RR) lag für ACR20, ACR50 bzw. ACR70 bei 1,40 (95 %-Konfidenzintervall, 95 %-KI: 1,13–1,74); 2,09 (95 %-KI: 1,67–2,61) bzw. 2,24 (95 %-KI: 1,53–3,28). Die Ergebnisse der Metaanalyse zeigten, dass keine statistische Signifikanz in Bezug auf Nebenwirkungen vorlag (1,06 (95 %-KI: 0,92–1,23)). Die Kombinationstherapie ist eine wirksame, sichere und wirtschaftliche Behandlungsoption für Patienten, die nicht gut auf Methotrexat allein ansprechen oder für Patienten, die sich teure Biologika nicht leisten können, deren Wirksamkeit sich nicht bestätigt hat.

## Introduction

Rheumatoid arthritis (RA) is an inflammatory disease of synovial joints that affects approximately 1% of the population [1]. In the absence of treatment, deteriorating joints can result in pain and stiffness, which limit physical function and lead to long-term disability [2]. RA is a potentially destructive disease that has a profound impact on functioning and quality of life among patients. At present, RA treatment remains a challenge for rheumatologists worldwide. Based on the course, disease activity, prognostic factors, and prior experience with disease-modifying antirheumatic drug (DMARDs) in the treatment of RA, the American College of Rheumatology (ACR) 2015 guidelines recommend the use of traditional antirheumatic agents and biological DMARDs. If methotrexate (MTX) is not found to be effective, methotrexate in combination with other DMARDs such as biologics will be recommended. However, the use of biological DMARDs to treat RA is not suitable for all patients for various reasons, including complications, side effects, uncertain efficacy, and high costs that prevent their use. Therefore, a new anti-rheumatic drug combined with MTX is urgently needed in the treatment of RA, especially for drug switching and cost reduction. Iguratimod (IGU) is a new synthetic disease-modifying antirheumatic drug (small molecule), whereby its mechanism of action is still not completely clear [3]. At the molecular level, iguratimod inhibits the invasiveness of rheumatoid synovial fibroblasts by decreasing matrix metalloproteinase (MMP‑1 and MMP-3) production [4], and it also inhibits the activation of MAPKs and the NF-kappa B pathway in RANKL-induced osteoclastogenesis in RAW264.7 cells, which can prevent bone destruction [5]. The production of IgG, IgM, and IgA was significantly reduced in active RA patients who were treated with IGU compared with those treated with placebo in some studies [6, 7]. Therefore, IGU has been considered a clinically useful DMARD with a unique mechanism of action, and its improvement rate of the ACR20 was not lower than that of sulfasalazine in patients with active RA (57.7% compared with 63.1%) [8]. Although the available study population is mainly East Asian (Japan and China up to date), and with the consequence that transfer of results to other ethnicities is questionable, we still need this research to show the effect of IGU on RA for rheumatologists around the world. The current systematic review and meta-analysis aims to evaluate the efficacy and safety of IGU combined with MTX versus MTX alone for RA.

## Methods

Our study was conducted following a protocol registered in PROSPERO (CRD42020157711). The methods used in the review conformed to the established Preferred Reporting Items for Systematic Reviews and Meta-Analyses (PRISMA) guidelines [9].

### Inclusion criteria

The inclusion criteria included the following: 1) studies conducted in a human population aged >18 years; 2) studies in which all patients were diagnosed with RA based on the ACR or the European League Against Rheumatism (EULAR) classification criteria; 3) studies that evaluated the combination of IGU and MTX therapy in RA; 4) studies with outcome data including ACR20, ACR50, ACR70, and adverse events; 5) randomized placebo-controlled clinical trials or trials in which the treatment arm was compared with a control arm; and 6) studies that were available in all languages. The full text of potentially related studies was extracted, and the methods and results of the trial were reviewed. When necessary data could not be determined, every effort was made to contact the study authors.

### Exclusion criteria

The exclusion criteria included the following: 1) nonhuman studies (animal studies) and studies in children; 2) studies in which patients diagnosed with RA were treated with a combination of other DMARDs; 3) studies with a duration <3 months; 4) non-RCT studies and studies without a clear control arm or placebo arm; and 5) studies without end-of-trial outcomes (ACR20, ACR50, ACR70, and adverse events). The participant, intervention, comparison, outcome, and study design (PICOS) data are presented in Table 1.

**Table 1:** PICOS criteria for inclusion and exclusion of studies

Parameter	Inclusion criteria	Exclusion criteria
Participant	Studies conducted in a human population aged >18 years Studies in which all patients were diagnosed with RA based on ACR or EULAR classification criteria	Nonhuman studies (animal studies) and studies among children Studies in which patients diagnosed with RA were treated with a combination of other drugs (DMARDs)
Intervention	Studies regarding the combination of iguratimod and methotrexate	Studies with a duration <3 months
Comparison	Studies that included comparisons between treatment with placebo + MTX or MTX (alone)	Studies without a clear control arm or placebo arm
Outcome	Studies in which ACR20, ACR50, and ACR70 were used as the evaluation criteria and as efficacy indicators Studies with safety evaluation	Studies without end-of-trial outcome data (ACR20, ACR50, ACR70, and adverse events)
Study design	Randomized controlled trials with parallel designs Studies available in all languages	Observational studies (which were excluded from the meta-analysis but were reviewed), studies without a placebo or control arm, and editorials and opinion pieces

*ACR* American College of Rheumatology, *EULAR* European League Against Rheumatism, *MTX* methotrexate, *DMARDs* disease-modifying antirheumatic drugs

### Literature search strategy

Two independent investigators searched for original RCT studies related to the combination of IGU and MTX therapy in RA published before November 1, 2019, in PubMed, Cochrane Library, Embase, the China National Knowledge Infrastructure (CNKI), the Chinese Biomedical Literature Database (CBM), and WanFang Data using the medical subject header (MeSH) terms “Iguratimod” or “T-614” and “Methotrexate” or “Amethopterin” or “MTX” and “Rheumatoid Arthritis” or “Arthritis, Rheumatoid” or “RA.” Additionally, we searched clinical trial registry websites (http://www.ClinicalTrials.gov and http://www.chictr.org.cn) to identify trials that had been completed but not yet published, as well as all ongoing trials with available results and data. When multiple publications were found regarding the same trial, we used the latest or the most complete report of the trial. If it included complete information about the study design, participant characteristics, interventions, and outcomes, we also considered conference summaries. Additional RCTs were identified from the reference lists of relevant full-text articles retrieved.

### Data extraction

Data were extracted independently by two investigators (LJ‑C and YJ-Z). A third investigator (JY-L) resolved the differences between the two investigators to reach a consensus. Only the results of outcomes that had been prespecified in the protocol were extracted from the studies. The following study characteristics were extracted: study title, unique study name or ID, primary author, contact information, publication type, funding and conflicts of interest, year the study was initiated and completed, country of origin of the study, study setting, study design (e.g., multicenter or single center), and study inclusion and exclusion criteria. The following study patient characteristics were extracted: total number of participants screened for the study, number of participants randomized into each study arm, and participant age, sex, and race/ethnicity. The following intervention and comparison group characteristics were collected: all information provided by the study for the regimen (therapeutic agent name, dose, route, frequency, and total duration of administration). The study outcomes were summarized. The adverse event definitions and the total number and individual number of events experienced were also extracted. Screening of the data extraction forms was completed to identify the missing or extraneous fields and to facilitate the most effective extraction process (LJ‑C and YJ-Z).

### Assessment of risk of bias

We assessed the methodological quality of the included trials using the Cochrane Collaboration tool. Studies were graded as having a “low risk”, “high risk,” or “unclear risk” of bias across the seven specified domains [10]. We also used the seven-point Jadad scale, which includes randomization, double-blinding, and sequential removal of one study at a time, a practice that is in agreement with the methods of other meta-analyses performed in this context [11]. Assessments were stored and managed in RevMan 5.3 (The Nordic Cochrane Centre, The Cochrane Collaboration, Copenhagen, Danmark; 2014).

### Data synthesis

For continuous data, the mean difference (MD) was determined using the DerSimonian and Laird method if the same scale was used across studies [12]. Results for dichotomous data were provided as relative risks and analyzed using the Mantel–Haenszel method. We examined heterogeneity in results across studies using Cochrane’s Q statistic, and inconsistency was quantified with the I^2^ statistic (100% × (Q-df) /Q), which represents the percentage of total variation across studies that is attributable to heterogeneity rather than chance [13]. We considered a *p*-value of less than 0.10 as indicative of substantial heterogeneity. When substantial heterogeneity was not observed, the pooled estimate calculated with the fixed-effects model was reported using the inverse variance method. When substantial heterogeneity was observed, the pooled estimate calculated with the random-effects model was reported using the DerSimonian and Laird method [12], which considers both within-study and between-study variation. Publication bias was evaluated via funnel plots (i.e., plots of study results against precision) and quantified with Begg’s and Egger’s tests [14, 15]. However, if there were fewer than 10 studies included in the systematic review, funnel plot analysis was not performed. A two-tailed *p*-value of less than 0.05 was considered statistically significant. Statistical analyses were performed using Review Manager (RevMan) 5.3. Meta-regression and publication bias analyses were performed using Stata version 14 software (StataCorp., College Station, TX, USA).

## Results

### Search results

**Fig. 1:**
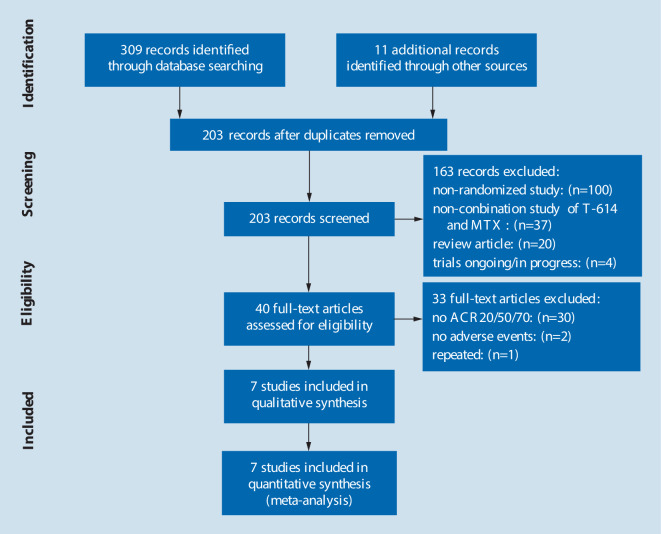
A flowchart of the literature search and selection process. *MTX* methotrexate, *ACR* American College of Rheumatology

All titles and abstracts found in the systematic review were independently screened by two researchers (LJ‑C, YJ-Z). A third investigator (JY-L) resolved the differences among two investigators to reach a consensus. Our search strategy yielded a total of 320 publications potentially related to the combination of IGU and MTX therapy for RA. After removing duplicates, a total of 203 studies were identified. Following screening of the titles and abstracts, 163 manuscripts did not fulfill our inclusion criteria and were excluded, leaving 40 selected manuscripts. After subsequent screening, an additional 33 were excluded: 30 randomized controlled trials (RCTs) did not include the ACR20/50/70, 2 RCTs did not report adverse events, and 1 RCT was repeated in more than one bibliographic source. After the selection process, a total of 7 RCTs consisting of 665 participants with 368 participants in the active arm and 297 in the placebo arm were included in the meta-analysis [1, 16–21]. A Preferred Reporting Items for Systematic Reviews and Meta-Analyses flow diagram is included in Fig. 1 to illustrate the number of publications included and excluded during each phase of screening.

### Study quality and risk assessment

**Fig. 2:**
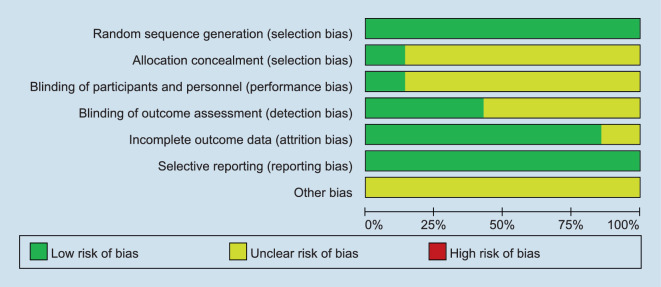
Risk of bias in the seven included studies

**Fig. 3:**
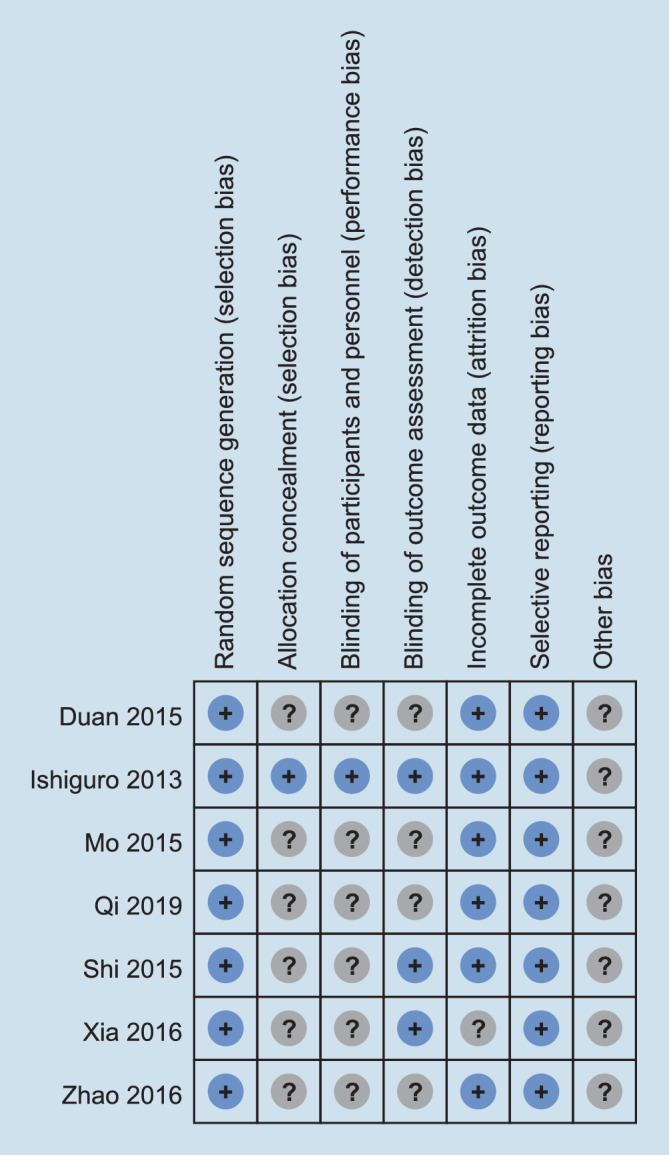
Summary of the risk bias in the seven included studies. *plus sign* low risk of bias, *question mark* unclear risk of bias

The included studies were published between 2013 and 2019. All included trials were randomized, with six phase II trials and three phase I trials. All seven trials were published as full manuscripts. Using the Cochrane Collaboration tool for risk of bias classification, we found the quality of the included studies to be generally good and fair (Figs. 2 and 3). Trials were also ranked for Jadad score (Table 2). The Jadad score of the included studies ranged from 3 to 4. The overall quality of the included studies was high.

**Table 2:** Baseline characteristics of the included trials in final analysis

Author, year	Design	Country	Study period	Eligible population	Primary outcome	Other outcome(s)	Jadad score
Duan et al. 2015 [17]	RCT, NR (blinding), single center	China	January 2013 to December 2013	2010 ACR and EULAR, not treated with any anti-rheumatism medicine or biological agents prior to enrollment and treatment	ACR20, TJC, SJC	ACR50, ACR70, HAQ, DAS28, ESR, CRP, APRs, SDAI, VAS (PAP, PGA, PhGA)	3
Ishiguro et al. 2013 [16]	RCT double-blind, placebo-controlled, multicenter	Japan	August 2009 to February 2011	Active RA patients (<10 years) based on 1987 ACR criteria, aged 20 to 70 years	ACR20 at week 24 or LOCF	TJC, SJC, DAS28, HAQ-DI, IgG, IgM, IgA, RF, VAS (PAP, PGA, PhGA), ESR, CRP	4
Xia et al. 2016 [1]	RCT, investigator blinding, single center	China	January 2013 to February 2014	Active RA patients based on 1987 ACR criteria being treated with traditional DMARDs	ACR20/50/70 at week 24	Morning stiffness, TJC, SJC, VAS (PAP, PGA, PhGA), ESR, CRP, DAS28-ESR, DAS28-CRP, HAQ	3
Qi et al. 2019 [18]	RCT, NR (blinding), single center	China	January 2015 to June 2018	Active RA patients based on 2012 ACR criteria, aged 25 to 65 years	ACR20 at week 24	TJC, SJC, DAS28, HAQ, VAS (PAP PGA PhGA), ESR, CRP	3
Shi et al. 2015 [20]	RCT, NR (blinding), single center	China	January 2013 to December 2013	Active RA patients based on 2010 ACR and EULAR criteria, aged >18 years, no history of using traditional DMARDs and biological agents	ACR20/50/70 at week 24	TJC, SJC, DAS28, HAQ, VAS (PAP, PGA, PhGA), ESR, CRP, SDAI	3
Mo et al. 2015 [19]	RCT, NR (blinding), single center	China	January 2013 to December 2014	Active RA patients based on 2010 ACR and EULAR criteria, aged >18 years	ACR20/50/70 at week 12	Morning stiffness, TJC, SJC, DAS28, HAQ, VAS (PAP, PGA, PhGA), ESR, CRP, RF, anti-CCP	3
Zhao et al. 2016 [21]	RCT, NR (blinding), single center	China	June 2013 to June 2015	Active RA patients based on 1987 ACR criteria, aged >18 years	ACR20/50/70 at week 24	TJC, SJC, DAS28, HAQ, VAS (PAP, PGA, PhGA)	3

*NR* not reported, *RCT* randomized controlled trial, *ACR* American College of Rheumatology, *EULAR* European League Against Rheumatism, *MTX* methotrexate, *DMARDs* disease-modifying anti-rheumatic drugs, *RA* rheumatoid arthritis, *LOCF* last observation carried forward, *TJC* tender joint count, *SJC* swollen joint count, *VAS* visual analog Scale, *PAP* patient’s assessment of pain, *PGA* patient global assessment, *PhGA* physician global assessment, *DAS28* Disease Activity Score 28, *HAQ* Health Assessment Questionnaire, *ESR* erythrocyte sedimentation rate, *CRP* C-reactive protein, *CCP* cyclic citrullinated peptides, *K* rheumatoid factors

### Baseline characteristics of the included studies

Table 2 summarizes the baseline characteristics of the included studies. All the studies were RCTs; five studies did not report the blinding methods, one reported a double-blind, placebo-controlled design [16], and one study applied blinding methods to the investigators [1]. Six trials were from China and were single-center studies, and the remaining trial was from Japan and was a multicenter study [16]. All studies were conducted between 2013 and 2018, often for more than a year. Patients participating in these trials followed eligibility criteria determined by each unique trial, which usually included active RA patients older than 18 years and were based on ACR or EULAR criteria. Three trials [16–18] reported that the primary efficacy endpoint was the rate at which patients (the full analysis set) achieved ACR20 at week 24 or last observation carried forward (LOCF), and the rate of ACR20/50/70 was reported in the other four studies. Other outcomes are detailed in Table 2.

### Characteristics of participants, interventions, and comparator details in the included studies

The characteristics of the participants, interventions, and comparator details in the included studies are presented in Table 3. One study [1] did not report age and sex characteristics for each group (only total age averages and sex ratios were reported), and complete age and sex characteristics were obtained for one study [21] by contacting the authors. The average age of the participants was similar across all studies. The iguratimod dose used in the study [16] was 25 mg once daily (QD) (0–4 weeks) and 25 mg twice daily (BID) (5–24 weeks), and the remaining six studies were 25 mg BID. The use of the MTX dose in three studies was phased using 10 mg/week (0–4 weeks) and 12.5 mg/week (5–24 weeks) [17, 20], and 7.5–10 mg/week (0–4 weeks) and 10 mg/week (5–24 weeks) [18]. However, there was no phased administration in four studies, including 6 or 8 mg/week [16], 10 mg/week [1, 21], and 15 mg/week [19]. It is worth noting that four studies added complementary drugs. Two studies [1, 20] allowed the use of one NSAID (0.2 g celecoxib capsule, two times a day, oral) and (or) a small dose of a glucocorticoid (prednisone 7.5 mg/d or 10 mg/d), and two studies allowed the use of folic acid 5 mg/week [16] or folic acid 10 mg/week [18].

**Table 3:** Intervention, comparators, and patient characteristics in evaluated studies

Author, year	Age (years)	Male/female	Patients enrolled (*n*)	Intervention (*n*)	Intervention details	Comparators (*n*)	Comparators details	Treatment duration	Patients for analysis
Duan et al. 2015 [17]	48.9 ± 12.2 48.4 ± 10.2	8/22 10/20	60	30	Celecoxib 0.4 g/day (0.2 g twice daily) and/or prednisone (7.5 mg/day) T‑614 50 mg/day (25 mg twice daily) MTX 10 mg/week first 4 weeks and at 12.5 mg/week later 20 weeks	30	Celecoxib 0.4 g/day (0.2 g twice daily) and/or prednisone (7.5 mg/day) MTX 10 mg/week first 4 weeks and at 12.5 mg/week later 20 weeks	24 w	30/30
Ishiguro et al. 2013 [16]	54.8 ±9.9 53.5 ± 10.0	30/134 70/18	252	164	Iguratimod 25 mg/day 0–4 weeks (25 mg once daily) and 50 mg/day for 5–24 weeks (25 mg twice daily) MTX 6 or 8 mg/week Folic acid 5 mg/week	88	MTX 6 or 8 mg/week Folic acid 5 mg/week	24 w	164/88
Xia et al. 2016 [1]	Total mean (SD) 46.63 ± 10.61	24/107	150	50	Iguratimod (25 mg, twice daily) plus MTX (10 mg once a week)	50/50	Iguratimod (25 mg, twice daily)/ MTX (10 mg once a week)	24 w	44/49
Qi et al. 2019 [18]	NR	NR	120	40	50 mg/day of iguratimod (25 mg twice daily) MTX 7.5–10 mg/week 0–4 weeks and folic acid at a dose of 10 mg/week	40/40	50 mg/day of iguratimod (25 mg twice daily)/ MTX 7.5–10 mg/week 0–4 weeks and folic acid at a dose of 10 mg/week	24 w	40/40
Shi et al. 2015 [20]	Total mean 48.7	Total 18/42	60	30	MTX 10 mg/week 0–4 weeks, 12.5 mg/week 5–24 weeks 50 mg/day of iguratimod (25 mg twice daily) All patients were allowed to use one NSAID (0.2 g of the celecoxib capsule, two times a day, oral) and (or) a small dose of a glucocorticoid (prednisone 10 mg/d)	30	MTX 10 mg/week 0–4 weeks, 12.5 mg/week 5–24 weeks All patients were allowed to use one NSAID (0.2 g of the celecoxib capsule, two times a day, oral) and (or) a small dose of a glucocorticoid (prednisone 10 mg/d)	24 w	30/30
Mo et al. 2015 [19]	31.8 ± 8.5 31.9 ± 8.6	8/22 9/21	60	30	Iguratimod (25 mg, twice daily) plus MTX (15 mg once a week)	30	MTX (15 mg once a week)	12 w	30/30
Zhao et al. 2016 [21]	NR	NR	90	30	Iguratimod (25 mg, twice daily) plus MTX (10 mg once a week)	30/30	Iguratimod (25 mg, twice daily)/ MTX (15 mg once a week)	24 w	30/30

*SD* standard deviation, *NR* not reported, *ACR* American College of Rheumatology, *EULAR* European League Against Rheumatism, *MTX* methotrexate, *DMARDs* disease-modifying anti-rheumatic drugs, *RA* rheumatoid arthritis, *NSAIDs* non-steroidal anti-inflammatory drugs, *GC* glucocorticoid, *w* weeks

### Efficacy of iguratimod combined with methotrexate

#### ACR20/50/70

**Fig. 4:**
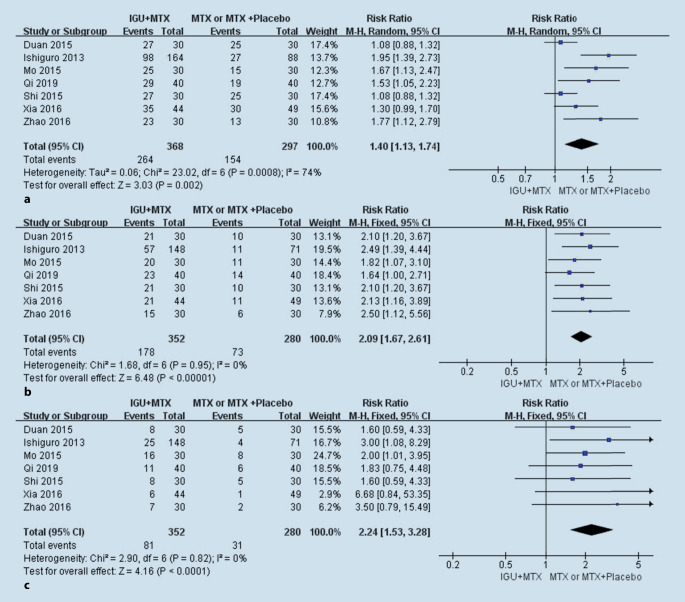
Comparison of ACR20 (**a**), ACR50 (**b**), and ACR70 (**c**) after treatment between the IGU + MTX group and the MTX group. *IGU* iguratimod, *MTX* methotrexate, *M‑H* Mantel–Haenszel method, *Random* random method, *CI* confidence interval

All seven studies compared the ACR, namely the ACR20, ACR50, and ACR70 between the IGU + MTX group and the MTX/MTX + placebo group. The heterogeneity (I^2^) values of the ACR20, ACR50, and ACR70 were 0.74 (*p* = 0.0008), 0 (*p* = 0.95), and 0 (*p* = 0.82), respectively; therefore, the random-effects model was used for ACR20 and the fixed-effects model was used for ACR50 and ACR70. The ACR was better for the IGU + MTX group, with a pooled relative risk (RR) for ACR20, ACR50, and ACR70 of 1.40 (95% CI 1.13–1.74), 2.09 (95% CI 1.67–2.61), and 2.24 (95% CI 1.53–3.28), respectively (Fig. 4).

#### DAS28

**Fig. 5:**
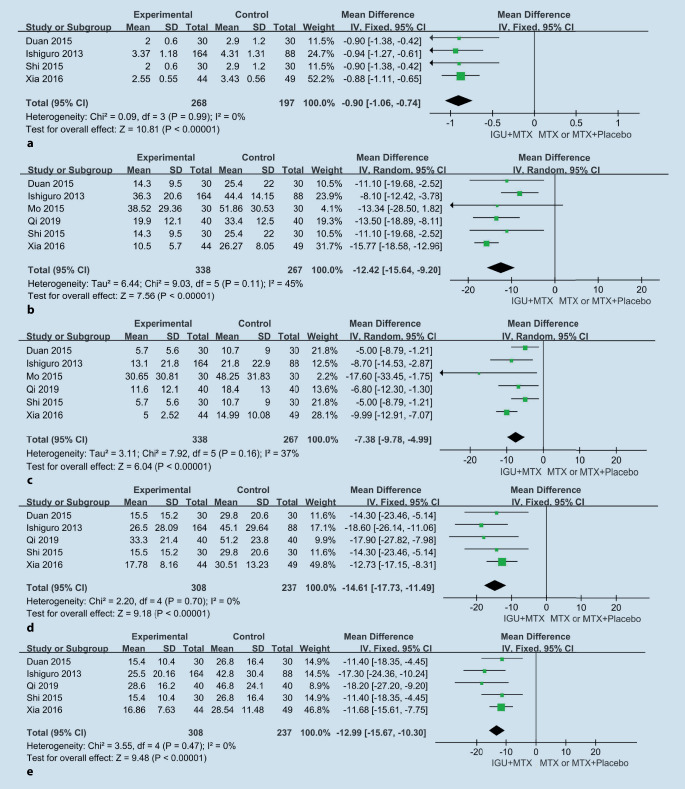
Comparison of DAS28 (**a**), ESR (**b**), CRP (**c**), VAS (PGA) (**d**), and VAS (PhGA) (**e**) after treatment between the IGU + MTX group and the MTX group. *SD* standard deviation, *IV* inverse variance methods, *IGU* iguratimod, *MTX* methotrexate, *CI* confidence interval

Four studies [1, 16, 17, 20] with a total of 465 participants were included in this meta-analysis (Fig. 5a). The heterogeneity was minimal (I^2^ = 0%), with no statistical significance (*p* = 0.99). Thus, the fixed-effects model was also used. The results demonstrated a significant decrease in DAS28 (−0.90 [95% CI −1.06–−0.74]). All the included studies showed that the combined therapy had a good effect with statistical significance.

#### ESR and CRP

Six studies [1, 16–20] that evaluated ESR and CRP levels at the end of treatment were included in the meta-analysis. The heterogeneity was moderate (ESR: I^2^ = 45%, *p* = 0.11; CRP: I^2^ = 37%, *p* = 0.16). The random-effects model was used. On pooled analysis (Fig. 5b, c), after treatment, the mean decreases in the levels of ESR and CRP were −12.42 mm/h (95% CI −15.64 to −9.20) and −7.38 mg/L (95% CI −9.78 to −4.99), respectively. All the included studies showed that the combined therapy had a statistically significant positive effect.

#### HAQ, TJC, SJC, and VAS (PAP, PGA, and PhGA)

Five studies [1, 16–18, 20] that evaluated HAQ, TJC, SJC, and VAS (PAP, PGA, and PhGA) levels at the end of treatment were included in the meta-analysis. The heterogeneity of HAQ, TJC, SJC, and VAS (PAP) was high (HAQ: I^2^ = 88%, *p* < 0.001; TJC: I^2^ = 76%, *p* = 0.002; VAS [PAP]: I^2^ = 83%, *p* < 0.001; and SJC: I^2^ = 90%, *p* < 0.001). However, the heterogeneity of VAS (PGA) and VAS (PhGA) was minimal (VAS [PGA]: I^2^ = 0%, *p* = 0.70; VAS [PhGA]: I^2^ = 0%, *p* = 0.47), and the fixed-effects model was used. On pooled analysis (Fig. 5d, e), after treatment, the mean decreases in the levels of VAS (PGA) and VAS (PhGA) were −14.61 (95% CI −17.73 to −11.49) and −12.99 (95% CI −15.67 to −10.30), respectively. Due to variability in the outcomes of HAQ, TJC, SJC, and VAS (PAP), meta-analysis was not feasible. However, in the included studies, there were statistically significant differences between the groups for HAQ, TJC, SJC, and VAS (PAP), which might suggest that IGU could possibly decrease HAQ, TJC, SJC, and VAS (PAP).

### The safety of combination therapy

**Fig. 6:**
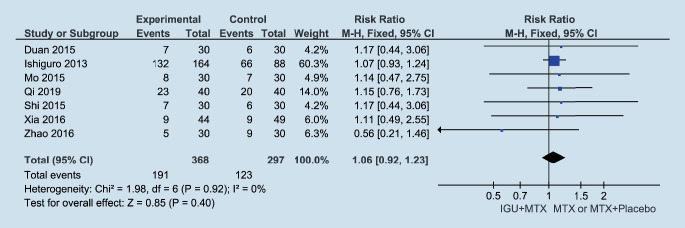
Analysis of the adverse events in the IGU + MTX group and the MTX group in the treatment of RA. *IGU* iguratimod, *MTX* methotrexate, *M‑H* Mantel–Haenszel method, *CI* confidence interval

All seven studies that evaluated adverse events were included in the meta-analysis (Fig. 6). The heterogeneity was minimal (I^2^ = 0%) without statistical significance (*p* = 0.92). The results demonstrated that there was no statistical significance in adverse events (1.06 [95% CI 0.92, 1.23]). The pooled RRs were generated by a fixed-effects model (Table 4). Combination therapy reported more leukopenia (16% vs. 13%); respiratory, thoracic, and mediastinal disorders (20% vs. 18%); increased β2-microglobulin (11% vs. 3%); and decreased blood iron (16% vs. 13%), but smaller increases in transaminase (20% vs. 26%) and gastrointestinal disorders (13% vs. 16%). There were no significant differences between the IGU + MTX and MTX /MTX + placebo groups for all adverse events, except increases in β2-microglobulin (RR: 4.31 [95% CI 1.32, 14.01]). One study [1] reported the adverse event of higher White Blood Cell (WBC) (2% vs. 3%; *p* > 0.05), one study[18] reported the adverse event of headache (3% vs. 3%; *p* > 0.05), and two studies [17, 20] reported the adverse event of dental ulcer (0% vs. 2%; *p* > 0.05).

**Table 4:** Adverse events (>5%) reported in the included studies

Categories of adverse events	IGU + MTX	MTX /MTX + Placebo	Risk ratio (95% CI)
Leukopenia	35 (16%)	16 (13%)	1.18 (0.68, 2.04)
Increment in transaminase	45 (20%)	31 (26%)	0.78 (0.52, 1.17)
Gastrointestinal disorders	28 (13%)	19 (16%)	0.79 (0.46, 1.36)
Respiratory, thoracic, and mediastinal disorders	44 (20%)	22 (18%)	1.08 (0.68, 1.71)
β2-microglobulin increased	24 (11%)	3 (3%)	4.31 (1.32, 14.01)
Blood iron decreased	35 (16%)	16 (13%)	1.18 (0.68, 2.04)

*IGU* iguratimod, *MTX* methotrexate, *CI* confidence interval

### Subgroup and sensitivity analysis

**Fig. 7:**
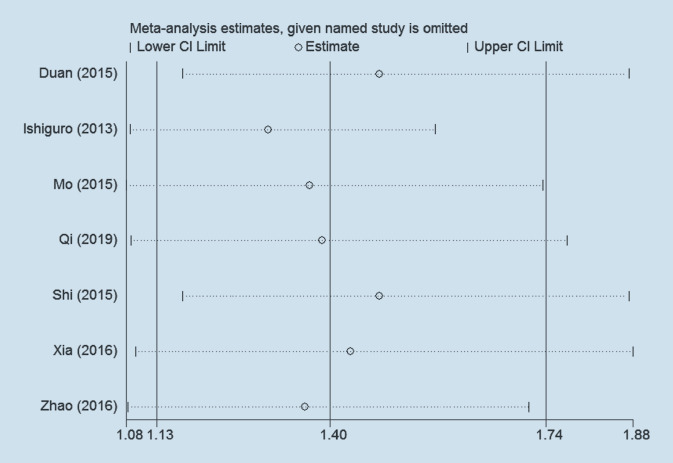
Sensitivity analysis of ACR20 after treatment between the IGU + MTX group and the MTX group. *MTX* methotrexate, *IGU* iguratimod, *CI* confidence interval

Due to the high heterogeneity of ACR20, we first checked whether the original data included in the study were correct and whether the method of extracting the data was correct. However, the whole process was normal. We compared the results between the fixed-effects model and the random-effects model for ACR20, and the RR was 1.47 (95% CI 1.30, 1.67) vs. 1.40 (95% CI 1.13, 1.74) in the outcome of ACR20. The conclusion of a favorable effect persisted even using different models. Furthermore, sensitivity analysis performed by Stata did not indicate alterations in the results (RR) by sequentially eliminating individual studies, suggesting that no single study significantly contributed to the heterogeneity of ACR20 (Fig. 7). However, we then performed sensitivity analysis of the outcome of ACR20 by eliminating individual studies from the meta-analysis model in RevMan, the most prominent of which was the study by Ishiguro et al. [16]. After excluding this study, the heterogeneity decreased from 74 to 59%, and the RR decreased from 1.40 (95% CI 1.13, 1.74) to 1.30 (95% CI 1.08, 1.57); however, the heterogeneity remained high and the RR was still significant, suggesting that this study may be a cause of the source of heterogeneity. Next, we carried out meta-regression and subgroup analysis of language, ACR standard, MTX phase, and complementary drugs. Meta-regression for language, ACR standard, MTX phase, and complementary drugs was not significant (*p* = 0.429, *p* = 0.923, *p* = 0.114, *p* = 0.378, respectively), indicating that none of these four covariates were the source of heterogeneity. Next, we carried out subgroup analyses of language, ACR standard, MTX phase, and complementary drugs (Table 5), although the outcome of meta-regression was not significant. Subgroup analysis by language revealed that there was statistical significance in the Chinese group (RR = 1.435, 95% Cl [1.058, 1.947], *p* = 0.02), but the English group showed the opposite result (RR = 1.162, 95% Cl [0.959, 1.407], *p* = 0.125). With respect to subgroup analysis by ACR standard, results for both the 1987 ACR standard (RR = 1.611, 95% CI [1.075, 1.917], *p* = 0.014) and no 1987 ACR standard (RR = 1.250, 95% CI [0.997, 1.569], *p* = 0.044) groups suggested obvious statistical significance. According to subgroup analysis by MTX phase, significant RR was found in the no MTX phase group (RR = 1.304, 95% CI [1.085, 1.567], *p* < 0.001), whereas no significant RR was reported in the two MTX phase group (RR = 1.154, 95% CI [0.95, 1.398], *p* = 0.145). Subgroup analysis by complementary drugs indicated that the used group (RR = 1.154, 95% CI [0.952, 1.398], *p* = 0.145) suggested no statistical significance, and the not-used group (RR = 1.47, 95% CI [1.205, 1.794], *p* = 0.011) marked a favorable effect of combination therapy.

**Table 5:** Pooled RRs for ACR20 according to subgroup analysis

Subgroup analysis		No of patients	No. of studies	Model	RR (95% CI)	*P*-value	Heterogeneity	
							I^2^	*P*-value
Language	*Chinese*	260	4	Random	1.435 (1.058, 1.947)	0.02	68.5	0.023
	*English*	405	2	Fixed	1.162 (0.959, 1.407)	0.125	27.2	0.241
ACR standard	*1987 ACR*	405	2	Fixed	1.436 (1.075, 1.917)	0.014	27.0	0.242
	*No 1987 ACR*	260	4	Random	1.250 (0.997, 1.569)	0.044	64.0	0.039
MTX phase	*Two phase*	200	3	Random	1.154 (0.952, 1.398)	0.145	46.9	0.152
	*Not phased*	465	3	Fixed	1.304 (1.085, 1.567)	<0.001	0	0.387
Complementary Drugs	*NSAIDs or (and) GC or folic acid*	120	3	Random	1.154 (0.952, 1.398)	0.145	46.9	0.152
	*Not used*	213	3	Fixed	1.470 (1.205, 1.794)	<0.001	0	0.387

*ACR* American College of Rheumatology, *EULAR* European League Against Rheumatism, *MTX* methotrexate, *DMARDs* disease-modifying anti-rheumatic drugs, *RA* rheumatoid arthritis, *NSAIDs* non-steroidal anti-inflammatory drugs, *GC* glucocorticoid, *RR *relative risk, *CI *confidence interval

### Publication bias

No evidence of publication bias was detected for the RR of ACR20, ACR50, or ACR70 by either Begg’s or Egger’s test (RR of ACR20 Begg’s *p* = 0.51 and Egger’s *p* = 0.62; RR of ACR50 Begg’s *p* = 0.29 and Egger’s *p* = 0.34; RR of ACR70 Begg’s *p* = 0.176 and Egger’s *p* = 0.065).

## Discussion

To the best of our knowledge, this is the first English systematic review and meta-analysis to compare the efficacy and safety of IGU combined with MTX in RA. According to our results, IGU + MTX had a positive impact on efficacy and safety in patients with RA by both increasing ACR criteria values, namely ACR20, ACR50, and ACR70, and decreasing clinical indexes including DAS28, ESR, CRP, VAS (PGA), and VAS (PhGA). Due to the variability in the outcomes of HAQ, TJC, SJC, and VAS (PAP), meta-analysis was not feasible. However, in the included studies, there were statistically significant differences between the groups for DAS28, ESR, CRP, HAQ, TJC, SJC, and VAS (PAP), which might suggest that IGU + MTX could possibly decrease these indexes.

Our meta-analysis of the main outcomes showed that ACR was better for the IGU + MTX group, with pooled RRs for ACR20, ACR50, and ACR70 of 1.40 (95% CI 1.13–1.74), 2.09 (95% CI 1.67–2.61), and 2.24 (95% CI 1.53‑3.28), respectively. Because of the high heterogeneity of ACR20, we performed a series of procedures to search for the source of heterogeneity, including checking the original data and the method used for extracting data repeatedly; performing sensitivity analysis with Stata and RevMan, meta-regression, and subgroup analyses; and changing from the fixed-effects model to the random-effects model. The sensitivity analysis performed by RevMan identified one study [16] in particular. After excluding this study, the heterogeneity decreased from 74 to 59%, and RR decreased from 1.40 (95% CI 1.13, 1.74) to 1.30 (95% CI, 1.08, 1.57). Hence, we identified two distinguishing factors that might have caused this difference after examining the study carefully. First, the quality of this study was the highest among all included studies, which was mainly reflected in the following aspects: inclusion of the largest number of people (*n* = 252), the highest Jadad score (4 points), rigorous experimental design (4-week observation period and a 24-week double-blind treatment period), and the phased collection of data (0 weeks, 8 weeks, 16 weeks, and 24 weeks). However, the other six studies included a small number of people (*n* = 60 to 100) and had an average Jadad score (3 points). The above reasons may lead to greater heterogeneity in the meta-analysis, but this does not mean that the study did not meet the inclusion criteria. Second, when designing this study, the researchers assumed ACR20 response rates of 50% in the iguratimod group and 25% in the placebo group, with a sample size of 128 patients and 64 patients (randomization ratio of 2:1). However, ACR20 was higher than 50% in most of the RCT experimental groups and 25% in the placebo group, and most of the RCTs were designed at a randomization ratio of 1:1. These factors may have resulted in the ACR20 rate of this study being different from that of other studies and being one of the sources of heterogeneity.

Iguratimod is a novel DMARD for the treatment of RA [1, 5, 22–35], which suppresses tumor necrosis factor-alpha-induced production of interleukin (IL)-6, IL‑8, and monocyte chemoattractant protein 1 via inhibition of nuclear factor kappa B activation in cultured human synovial cells and human acute monocytic leukemia cells, which indirectly inhibits damage to osteoblasts [16, 34, 36, 37]. Moreover, IGU also can reduce immunoglobulin (Ig) production by acting directly on human B lymphocytes without affecting proliferation [38]. In addition, by reducing serum levels of tumor necrosis factor-alpha, IL-1-beta, IL-17, and IL‑6, IGU markedly enhances the therapeutic effect (synergistic effect) of the combined treatment [33, 38, 39]. Because of the different active mode of iguratimod and because the efficacy of the combined treatment of MTX + IGU is better than that of MTX + placebo, the combined treatment is an effective, safe, and economical treatment option for patients who do not respond well to MTX alone or for patients who cannot afford expensive biologics that have no confirmed efficacy.

The event rates of adverse effects were similar in the two groups. The only significant increase in adverse events was an increase in β2-microglobulin in one RCT [16]. Moreover, one study [1] reported the adverse event of higher WBCs (2% vs. 3%; *p* > 0.05), one study [18] reported the adverse event of headache (3% vs. 3%; *p* > 0.05), and two studies [17, 20] reported the adverse event of dental ulcer (0% vs. 2%; *p* > 0.05). Some studies have shown that peculiar hemorrhage (pulmonary alveolar hemorrhage, subcutaneous hemorrhage) was observed with concomitant use of IGU and warfarin [3, 39]. One study in vivo indicated that the mechanism by which IGU increases the anti-coagulation activity of warfarin is by modulating the production of a blood coagulation factor by the vitamin K cycle [40]. However, another study in vitro indicated that IGU is a potent direct inhibitor of CYP2C9-mediated warfarin 7‑hydroxylation [41]. Further studies should help identify and explore specific mechanisms. No deaths were reported in any of the included studies. Iguratimod combined with methotrexate appeared to be safe and tolerable for RA.

## Strengths and limitations

Our systematic review with meta-analysis has several strengths. We searched the major databases (including trials, gray literature, and unpublished data) with rigorous strategies. Two authors selected the articles independently, allowing a low probability that an important study was missed. The included studies were of high quality. Although the heterogeneity of the ACR20 was high, we performed a series of procedures to detect the source of heterogeneity, including checking the original data and the method of extracting data repeatedly; performing sensitivity analysis by Stata and RevMan, meta-regression, and subgroup analyses; and changing from the fixed-effects model to the random-effects model. Moreover, there was no significant publication bias. We evaluated all clinical indexes comprehensively, including DAS28, ESR, CRP, VAS (PAP), VAS (PGA), and VAS (PhGA). However, there were some limitations. Firstly, the strength of our research was compromised by the small number of trials[42]. Secondly, no phase III trials were available for analysis. Six trials were single-center China-based studies. Only one multicenter Japan-based study remained. Thirdly, the actual molecular targets of IGU are still unknown, which further studies should help to predict. The clinical studies on this drug are mainly short-term, there are no long-term clinical data for more than 3 years. Therefore, multicenter and long-term safety data and comparisons of the safety and effectiveness of IGU with other drugs are necessary. Furthermore, the largest and most rigorous RCT that had the greatest influence on our results was different from the other RCTs regarding the statistical analysis design. Additionally, the tests of publication bias had low power to detect a potential bias. In addition, the people we included in the study were all Asian (mainly Chinese and Japanese) and could be ethnically different. The impact is that our conclusions may not be generalizable to other populations such as Americans or Europeans. In our review, the adverse events of higher WBC, headache, and dental ulcer were reported in only one or two studies. Serious adverse events were reported in one study [16], with 5 patients in the IGU + MTX group (gastroduodenal ulcer, tendon rupture, carbon monoxide poisoning, interstitial lung disease, and retinal hemorrhage), and Li et al. reported a rare case of severe liver injury. Although no deaths were reported in any included studies, the safety of combination therapy requires careful monitoring of adverse events throughout iguratimod treatment for diseases.

## Conclusion

Overall, the meta-analysis of RCTs strongly suggests that iguratimod combined with methotrexate is more effective in treating RA than methotrexate alone. It is also worth noting that the incidence of adverse events associated with combination therapy is comparable to methotrexate alone, with the exception of some low-incidence events. Combination therapy is an effective, safe, and economical treatment option for patients who do not respond well to methotrexate alone or for patients who cannot afford expensive biologics that have no confirmed efficacy. Finally, more large multicenter randomized controlled trials, especially non-Asia-Pacific trials, are needed to produce more reliable conclusions.

## Abbreviations

ACR: American College of Rheumatology
CCP: Cyclic citrullinated peptides
CRP: C‑reactive protein
DAS28: Disease Activity Score 28
DMARDs: Disease-modifying antirheumatic drugs
ESR: Erythrocyte sedimentation rate
EULAR: European League Against Rheumatism
GC: Glucocorticoid
HAQ: Health Assessment Questionnaire
LOCF: Last observation carried forward
MTX: Methotrexate
NR: Not reported
NSAIDs: Non-steroidal anti-inflammatory drugs
PAP: Patient’s assessment of pain
PGA: Patient global assessment
PhGA: Physician global assessment
RCT: Randomized controlled trial
RF: Rheumatoid factors
SD: Standard deviation
SJC: Swollen joint count
TJC: Tender joint count
VAS: Visual analog Scale
